# Obinutuzumab in systemic lupus erythematosus: a real-world experience

**DOI:** 10.3389/fimmu.2025.1702550

**Published:** 2025-11-19

**Authors:** Chunmei Wu, Yinglu Wang, Shenshen Chen, Yanwei Lin, Fang Du, Xiaodong Wang, Sheng Chen, Liangjing Lu, Shuang Ye, Huihua Ding, Qiong Fu

**Affiliations:** 1 Department of Rheumatology, Renji Hospital, Shanghai Jiao Tong University School of Medicine, Shanghai, China; 2 Shanghai Immune Therapy Institute, Shanghai, China

**Keywords:** systemic lupus - erythematosus, obinutuzmab, biologics, real world study, B cell depletion

## Abstract

**Objectives:**

To describe the real-world efficacy and safety of obinutuzumab in a heterogeneous systemic lupus erythematosus (SLE) population, including severe or refractory manifestations.

**Methods:**

We retrospectively analyzed 56 SLE patients who received a single dose of obinutuzumab (1000 mg) at Renji Hospital, Shanghai Jiao Tong University School of Medicine between October 2021 and March 2024. Patients were followed for 48 weeks. Outcomes included the proportion of patients achieving definitions of remission in systemic lupus erythematosus (DORIS), Lupus Low Disease Activity State (LLDAS), clinical stable, and flare. Changes in SLEDAI-2K, anti-dsDNA, complement levels, B cell counts, glucocorticoid dose, and adverse events were assessed. Subgroup analyses were performed to address population heterogeneity.

**Results:**

At baseline, 35.7% of patients were newly diagnosed, 55.4% had relapsing/refractory disease, and 8.9% were on maintenance therapy. By week 48, 37.5% achieved DORIS remission, 32.1% LLDAS, and 19.6% remained clinically stable. Mean SLEDAI-2K decreased from 11.75 ± 9.31 (range: 0 to 46) to 1.45± 1.93 (range: 0 to 8). Among 15 lupus nephritis patients, 86.7% achieved complete renal response. Significant hematologic improvement was observed in autoimmune hemolytic anemia and thrombocytopenia. B cell counts declined rapidly and began to repopulate from week 36. Glucocorticoids tapered from 43.04 ± 18.19 (range: 0 to 60) to 8.95 ± 9.78 (range: 0 to 50) mg/day. Obinutuzumab was well tolerated, with infections observed in 14 cases (25.0%) and infusion reactions in 4 cases (7.1%).

**Conclusion:**

Obinutuzumab showed favorable efficacy and safety in SLE, including severe/refractory manifestations, suggesting potential benefits for difficult-to-treat patients.

## Introduction

Systemic lupus erythematosus (SLE) is a chronic autoimmune disease of unclear etiology that affects multiple vital organs, which is characterized by a relapsing-remitting course and is associated with significant morbidity and mortality (1). B cells play a central role in the pathogenesis of SLE. Although conventional treatments such as glucocorticoids and broad-spectrum immunosuppressants remain the cornerstone of therapy, their non-specific immunosuppressive effects often lead to substantial adverse events and suboptimal efficacy in certain patients. As a result, B cell–targeted therapy has emerged as a promising alternative strategy.

CD20, a transmembrane protein broadly expressed on B cells at various developmental stages, is a key therapeutic target for B cell depletion (2). Rituximab, a type I anti-CD20 antibody, primarily mediates complement-dependent cytotoxicity (CDC). Previous SLE trials of rituximab, such as EXPLORER and LUNAR, failed to meet their primary endpoints despite showing clinical signals. This outcome has been partly attributed to heterogeneity in trial design and patient selection, which may have limited the interpretability of the results (3, 4).

In contrast, obinutuzumab is a glycoengineered, humanized type II anti-CD20 IgG1 monoclonal antibody approved for the treatment of hematologic malignancies such as chronic lymphocytic leukemia and follicular lymphoma (5). Unlike rituximab, obinutuzumab targets a distinct epitope on CD20 and induces more potent direct cell death, antibody-dependent cellular cytotoxicity (ADCC), and phagocytosis, while CD20 internalisation is reduced (6). These mechanistic advantages may enable more consistent and durable B cell depletion. Supporting this, the phase II NOBILITY trial and the phase III REGENCY study demonstrated the efficacy and favorable safety profile of obinutuzumab in patients with lupus nephritis (LN), with the latter representing a milestone as the first anti-CD20 agent to meet its primary endpoint in a phase III trial in this indication (7, 8). Nonetheless, these studies were conducted exclusively in LN populations under highly controlled conditions, and differences in disease spectrum, background therapies, and outcome measures constrain the generalizability of their findings. Moreover, real-world data on obinutuzumab in SLE remain scarce, underscoring the need for further evaluation of its clinical effectiveness.

Therefore, this study aims to assess the efficacy and safety of obinutuzumab in a real-world cohort of patients with SLE over a 48-week follow-up period.

## Methods

This was a single-center retrospective study conducted at Renji Hospital, Shanghai Jiaotong University School of Medicine, involving patients diagnosed with systemic lupus erythematosus (SLE) according to the 2019 EULAR/ACR classification criteria (9), who received obinutuzumab between October 2021 and March 2024. Lupus nephritis (LN) was diagnosed according to the 2019 EULAR/ERA-EDTA recommendations (10). Kidney biopsy was performed in patients with significant urinary abnormalities or proteinuria ≥0.5 g/day when clinically feasible, and the pathological classifications were based on the 2003 ISN/RPS criteria (11). Thrombotic microangiopathy (TMA) was diagnosed based on renal biopsy findings when available or on compatible clinical features after excluding other secondary causes. Complement testing (C3, C4, CH50) was performed in all patients, while advanced complement assays were not routinely available. A total of 56 patients were included and followed for 48 weeks. The study design and reporting adhered to the STROBE guidelines, and a flow diagram summarizing patient screening, inclusion, and exclusion was shown in **Supplementary Figure S1**. Each patient received one single dose of obinutuzumab (1000 mg) on day 1, along with intravenous methylprednisolone (80 mg) administered on the same day. The use of concurrent immunosuppressants and other biologics was permitted during the follow-up period. The decision to employ sequential immunotherapy was individualized according to disease activity, organ involvement, and physician judgment, with the dual purpose of either providing enhanced control as part of baseline therapy or rescuing an inadequate response or relapse after obinutuzumab.

Clinical data were collected at baseline and at follow-up visits at weeks 4, 12, 24, 36, and 48. Immunological markers, including anti-dsDNA antibodies, complement levels, B cell counts, and immunoglobulin levels, were monitored serially.

Disease activity was assessed using the Systemic Lupus Erythematosus Disease Activity Index 2000 (SLEDAI-2K) (12). Treatment responses were categorized as follows: DORIS remission (as defined by the Definitions of Remission in SLE) (13); lupus low disease activity state (LLDAS) but excluding patients who had already achieved DORIS remission (14); clinical stable, a modified form of LLDAS that allows a prednisone dose of 7.5–10 mg/day (15); disease flare: an increase in the SLEDAI-2K score by ≥4 points compared to the previous visit or new active clinical manifestations requiring treatment adjustment (16); and persistent: SLEDAI-2K >4 and prednisone >10 mg/day at all assessed time points (17). Treatment efficacy of patients with LN was assessed based on complete renal response (CRR), partial renal response (PRR), and no renal response (NRR), in accordance with the 2024 KDIGO clinical practice guidelines for the management of LN (18). Safety was evaluated by recording the proportion of patients who experienced adverse events (AEs).

The study was approved by the Ethics Committee of Renji Hospital. Written informed consent was obtained from all participants prior to treatment.

Unless otherwise stated, continuous variables were presented as mean ± standard deviation (SD). Categorical variables were reported as proportions with 95% confidence intervals (CIs). Missing data were handled by pairwise deletion in longitudinal comparisons. Statistical analyses were performed using SPSS version 29.0.1.0 (171) (IBM) for statistical tests, and Prism version 10.3.0 (GraphPad Software) and R version 2024.12.1 + 563 (R Foundation for Statistical Computing) for data visualization. Paired t-tests (two-tailed) were used for comparisons, and P value < 0.05 was considered statistically significant.

## Results

**Figure 1:**
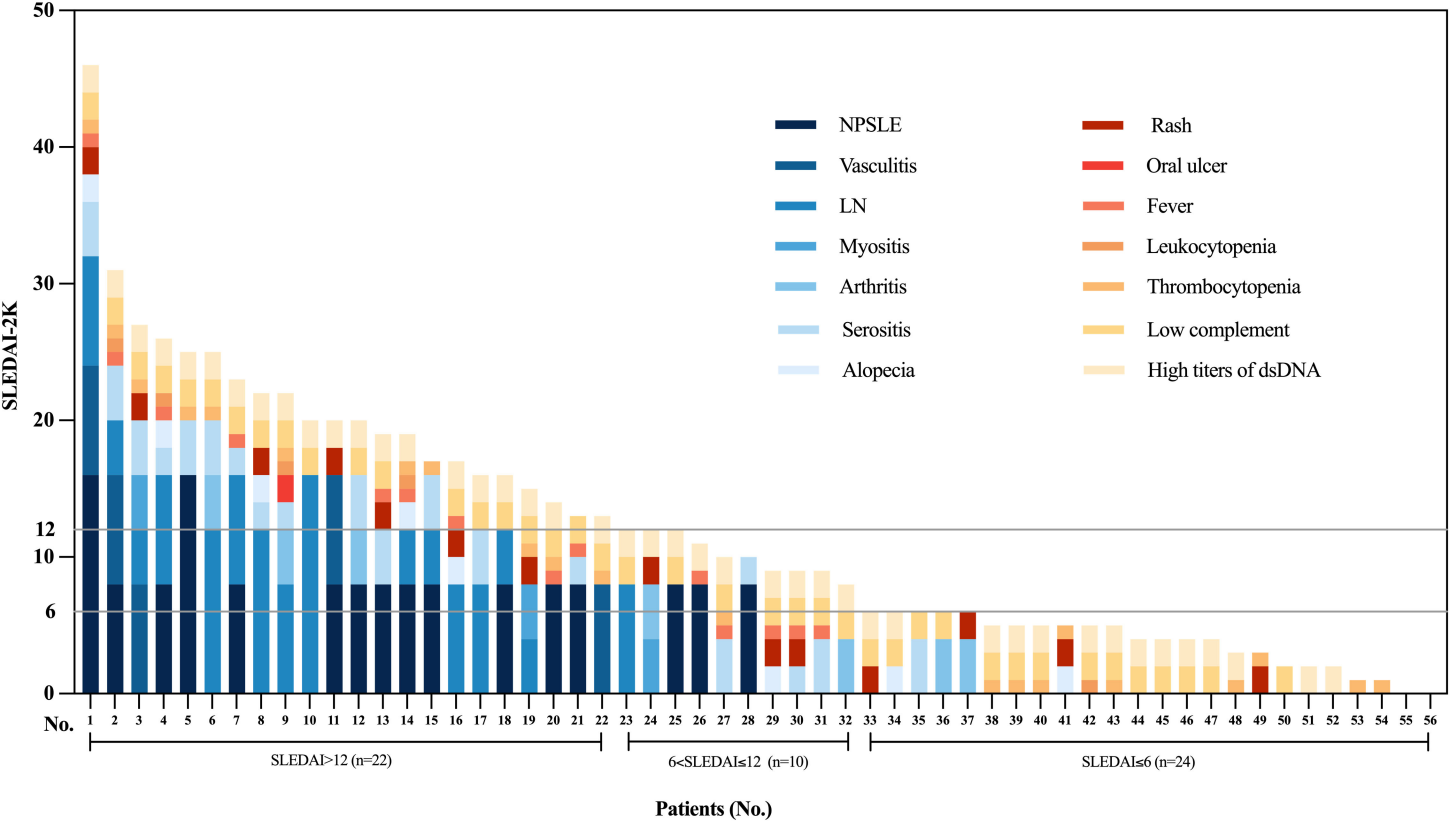
Baseline characteristics of SLE patients treated with obinutuzumab. The stacked bar chart displays individual patients’ SLEDAI-2K scores. Each bar represents a single patient and is stacked by individual SLEDAI-2K components. Patients are grouped by SLEDAI-2K score at baseline into three categories: SLEDAI ≤ 6 (n=24), 6<SLEDAI ≤ 12 (n=10), and SLEDAI>12 (n=22).

A total of 56 patients were involved in this study. The majority of the patients were females (80.3%), with the mean age at obinutuzumab treatment of 30.98 ± 12.45 (range: 13 to 65).years old. At the time of obinutuzumab administration, 20 (35.7%) patients were experiencing their first disease episode, 31 (55.4%) had relapsing or refractory disease, and 5 (8.9%) received obinutuzumab as part of maintenance therapy. Baseline disease activity was measured by a mean SLEDAI-2K score of 11.75 ± 9.31(range: 0 to 46), and a Physician’s global assessment (PGA) score of 1.66 ± 0.87(range: 0 to 3). Specifically, 24 patients (42.9%) had a SLEDAI-2K score ≤6, 10 patients (17.9%) between 6 and 12, and 22 patients (39.3%) >12 (**Figure 1**). The most common clinical manifestations included hematologic abnormalities (30 cases, 53.6%), mucocutaneous involvement (20 cases, 35,7%), neuropsychiatric lupus (18 cases, 32.1%), fever (15 cases, 26.8%), LN (15 cases, 26.8%), and musculoskeletal involvement (10 cases, 17.9%). Besides, patients with gastrointestinal vasculitis (5 cases, 8.9%) and TMA (4 cases, 7.1%) were also included in our study. Among the 15 patients with LN, 4 underwent renal biopsy before obinutuzumab initiation, and the pathological classifications were class III in 2 cases, class IV in 1 case, and class IV + V in 1 case. Biopsy-confirmed TMA was observed in one patient. Prior to obinutuzumab treatment, 85.7% of patients had received corticosteroids, 73.2% with hydroxychloroquine, 58.9% with traditional immunosuppressive agents and 26.8% with biologic agents (**Table 1**). When stratified by disease stage, new-onset patients exhibited the highest baseline disease activity. In contrast, relapsing/refractory and maintenance cases were characterized by longer disease histories and greater prior use of immunosuppressants or biologics (**Supplementary Table S1**). More detailed baseline features were summarized in **Table 1**; **Supplementary Table S1**.

**Table 1:** Baseline characteristics of SLE patients treated with obinutuzumab.

SLE Patients (n=56)	
Epidemiologic features	
Female, n (%)	45 (80.3)
Disease duration at first treatment, mean ± SD, (months)	48.52 ± 49.32 (0.3-168)
Disease state, n (%)	
New-onset	20 (35.7)
Relapsing	27 (48.2)
Refractory	4 (7.1)
Maintenance	5 (8.9)
Age at obinutuzumab treatment, mean ± SD, (years)	30.98 ± 12.45 (13-65)
Clinical features, n (%)	
Fever	15 (26.8)
Mucocutaneous involvement	20 (35.7)
Musculoskeletal involvement	10 (17.9)
Serositis	19 (33.9)
Hematologic abnormalities	30 (53.6)
Autoimmune hemolytic anemia	12 (21.4)
Hemoglobin level≦60g/L	3 (25.0)
Immune thrombocytopenia	15 (26.8)
Plate level≦30×10^9^/L	5 (33.3)
Leukocytopenia	5 (8.9)
Lupus nephritis	15 (26.8)
24-hour urine protein, mean ± SD, (g/24h)	2.28 ± 0.49 (0.67-6.09)
Serum creatinine, mean ± SD, (µmol/L)	83.83 ± 41.29 (32-185)
Neuropsychiatric lupus	18 (32.1)
Gastrointestinal vasculitis	5 (8.9)
Pulmonary hypertension	4 (7.1)
Thrombotic microangiopathy	4 (7.1)
Myocardial involvement	2 (3.6)
Antiphospholipid syndrome	
Deep venous thrombosis	7 (12.5)
Cerebral infraction	3 (5.4)
Pulmonary embolism	1 (1.8)
Serologic characterization	
WBC, mean ± SD, (×10^9^/L)	8.10 ± 4.29 (1.02-21.72)
Hb, mean ± SD, (g/L)	104.89 ± 26.06 (51.00-154.00)
PLT, mean ± SD, (×10^9^/L)	167.71 ± 120.57 (2.00-494.00)
C3, mean ± SD, (g/L)	0.65 ± 0.28 (0.19-1.34)
C4, mean ± SD, (g/L)	0.12 ± 0.10 (0.02-0.66)
IgA, mean ± SD, (g/L)	2.57 ± 1.26 (0.27-6.55)
IgM, mean ± SD, (g/L)	1.03 ± 0.70 (0.21-3.00)
IgG, mean ± SD, (g/L)	16.7 ± 8.9 (6.15-53.90)
Anti-dsDNA (ELASA), mean ± SD, (IU/mL)	167.90 ± 131.26 (22.46-505.06)
Anti-dsDNA (RIA), mean ± SD, (IU/mL)	51.66 ± 35.32 (4.96-100)
Antinuclear antibody positivity, n (%)	56 (100.0)
Anti-SSA positivity, n (%)	28 (50.0)
Anti-Ro52 positivity, n (%)	23 (41.1)
Anti-SSB positivity, n (%)	7 (12.5)
Anti-U1RNP positivity, n (%)	20 (35.7)
Anti-Sm positivity, n (%)	7 (12.5)
Anti-Rib-P positivity, n (%)	15 (26.8)
Anti-Histone positivity, n (%)	12 (21.4)
Antiphospholipid antibody positivity, n (%)	37 (66.1)
B lymphocyte levels	
B lymphocyte percentage, mean ± SD, (%)	17.76 ± 12.07 (1.62-44.98)
B lymphocyte count, mean ± SD, (cells/µL)	247.65 ± 293.74 (13.10-1268.40)
Disease activity	
SLEDAI-2K, mean ± SD	11.75 ± 9.31(0-46)
PGA, mean ± SD	1.66 ± 0.87 (0-3)
Previous treatments, n (%)	
Glucocorticoid	48 (85.7)
Hydroxychloroquine	41 (73.2)
Immunosuppressants	33 (58.9)
MMF	20 (35.7)
TAC	14 (25.0)
CTX	9 (16.1)
CsA	8 (14.3)
LEF	6 (10.7)
MTX	5 (8.9)
Tripterygium glycosides	1 (1.8)
AZA	1 (1.8)
Biologics	15 (26.8)
RTX	11 (19.6)
Belimumab	5 (8.9)
Telitacicept	2 (3.6)

SD, standard deviation; SEM, standard error of the mean; MMF, Mycophenolate mofetil; TAC, Tacrolimus; CTX, Cyclophosphamide; CsA, Cyclosporine A; LEF, Leflunomide; MTX, Methotrexate; AZA, Azathioprine; RTX, Rituximab; ELASA, Enzyme-Linked Immunosorbent Assay; RIA, Radioimmunoassay.

**Figure 2:**
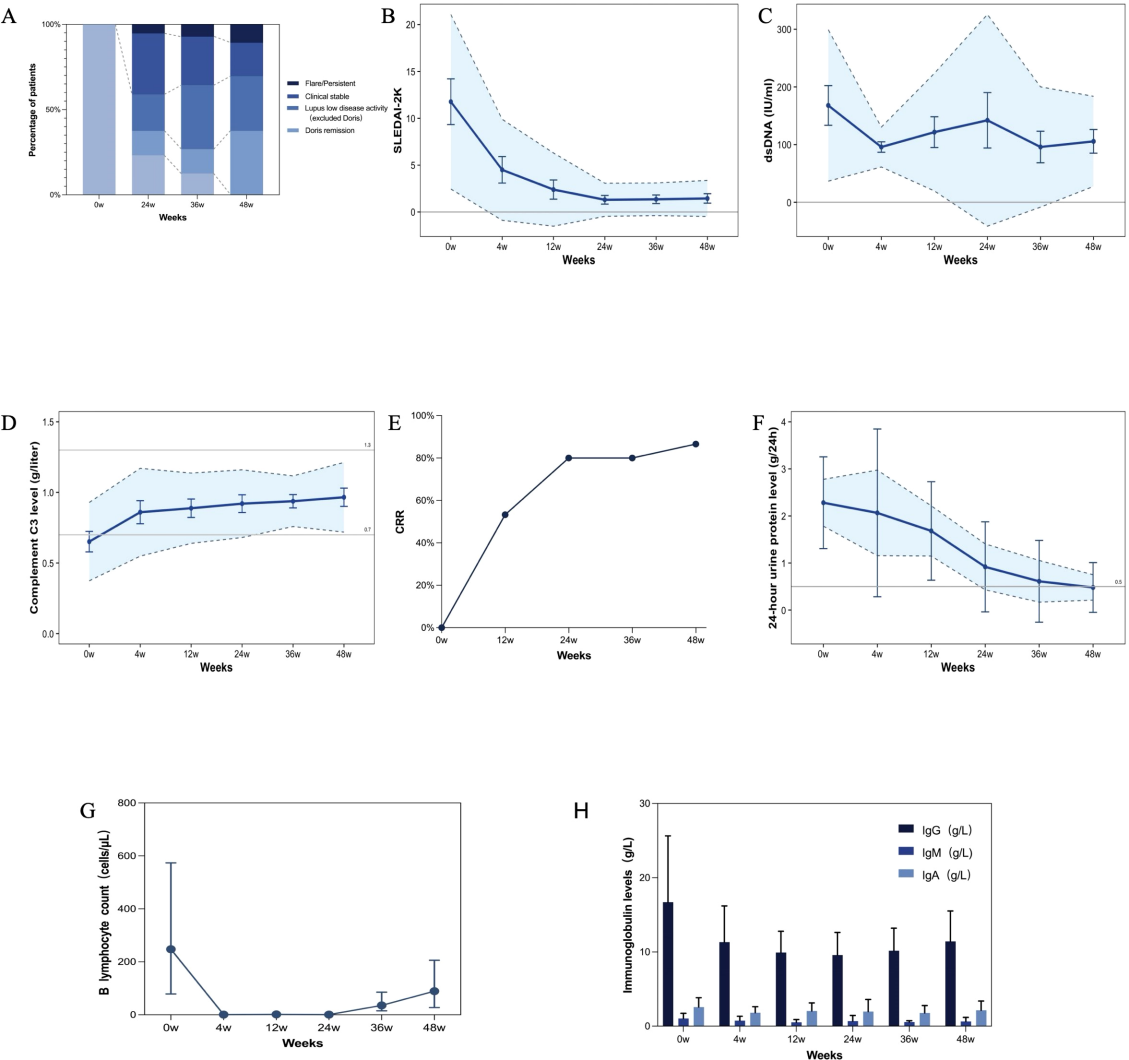
Clinical and serological improvements following obinutuzumab treatment in SLE patients. **(A)** Changes in disease states over time. **(B-D)** SLEDAI-2K Score **(B)**, anti-dsDNA antibody level **(C)** and complement C3 **(D)** changes during the 48-week follow-up. The shaded areas represent the standard deviation (SD), the error bars illustrate 95% confidence intervals (CIs), and the lines indicate the mean value at each time point. **(E)** Complete renal response (CRR) rate changes in patients with lupus nephritis during follow-up. CRR was achieved in 53.3% of patients at week 12, 80% at week 24, and 86.6% at week 48. **(F)** Changes in proteinuria before and after obinutuzumab treatment. The solid line represents the mean values, the error bars illustrate 95% confidence intervals (CIs), and the shaded area indicates the standard error of the mean (SEM). **(G)** Dynamics of B cell numbers during the 48-week follow-up. The error bars illustrate 95% confidence intervals (CIs). **(H)** Mean serum immunoglobulin level changes during follow-up. The shaded areas represent the standard deviation (SD), and the lines indicate the mean value at each time point.

At week 24, 14.3% (8 cases, 95% confidence interval [CI], 5.1 to 23.5) of patients achieved DORIS remission, 21.4% (12 cases, 95% CI, 10.8 to 32.0) reached LLDAS, and 35.8% (20 cases, 95% CI, 23.4 to 48.2) were clinically stable. By week 48, response rates improved to 37.5% (21 cases, 95% CI, 24.7 to 50.3) for DORIS remission, 32.1% (18 cases, 95% CI, 19.8 to 44.4) for LLDAS, and 19.6% (11 cases, 95% CI, 9.2 to 30.0) for clinical stable (**Figure 2A**). Mean SLEDAI-2K scores declined significantly from baseline, reaching 4.50 ± 5.39 at week 4 (95% CI, 3.09–5.91; range: 0 to 20) and 1.45 ± 1.93 at week 48 (95% CI, 0.94 to 1.96; range: 0 to 8) (**Figure 2B**). Subgroups analysis revealed that all three subgroups, especially the new-onset and relapsing/refractory groups, showed a rapid decrease in SLEDAI-2K scores, which were maintained at low levels through week 48 (**Supplementary Figure S3**). Besides, anti-dsDNA antibody levels decreased, while complement levels exhibited a notable upward trend throughout the follow-up period (**Figures 2C, D**; **Supplementary Figure S2A**). Disease flares occurred in five patients: one with rash at week 24, one with recurrent proteinuria at week 36, and three had flares at week 48 (presenting with fever and rash, fever with serositis, and recurrent NPSLE, respectively). Three patients had persistent disease activity (two with LN and one with refractory rash) (**Figure 2A**).

Encouraging clinical responses were observed in patients with major organ involvement. Among 15 patients with LN, the CRR rate at week 48 was 86.7% (95% CI, 69.5 to 100.0), and the overall renal response (ORR) reached 93.3% (95% CI, 79.6 to 100.0) (**Figure 2E**; **Supplementary Figure S2B**). Proteinuria decreased significantly from 2.28 ± 0.49 g/24h at baseline (range: 0.67 to 6.09g) to 0.27 ± 0.26 g/24h at week 48 (95% CI, 0.13 to 0.41; range: 0.05 to 4.23g) (**Figure 2F**), while serum creatinine levels remained stable throughout follow-up (**Supplementary Figure S2C**). The single patient who did not achieve a renal response had an eight-year disease history and had demonstrated resistance to multiple immunosuppressants, including cyclophosphamide, mycophenolate mofetil, methotrexate, leflunomide, and telitacicept. Among patients with hematologic involvement, significant improvements were observed following treatment: In 12 patients with autoimmune hemolytic anemia (AIHA), hemoglobin levels rose from 78.7 ± 19.7 g/L (range: 51 to 116) to 126.8 ± 21.1 g/L by week 48 (95% CI, 113.6 to 140.0; range: 90 to 171); Similarly, in 15 patients with immune thrombocytopenia (ITP), platelet counts increased from 71.8 ± 66.2×10^9^/L (range: 2 to 255) to 195.8 ± 77.3×10^9^/L (95% CI, 155.4 to 236.2; range: 78 to 367), indicating robust hematologic response (**Supplementary Figure S2D**).

Absolute B cell counts declined markedly from a baseline of 247.65 ± 292.97 cells/μL to nearly undetectable levels (mean <5 cells/μL) by week 4, remaining suppressed through week 24. Reconstitution began at week 36 (35.25 ± 55.84 cells/μL) and continued through week 48 (89.35 ± 102.43 cells/μL; 95% CI, 61.2–117.5). Most patients entered a state of peripheral B cell depletion during treatment, followed by gradual repopulation over time (**Figure 2G**). Notably, 38.2% (95% CI, 25.0 to 51.4) of patients still exhibiting B cell counts below 10 cells/μL at week 48. Serum IgG, IgA, and IgM levels also declined, consistent with the expected pharmacodynamic effects (**Figure 2H**).

Glucocorticoids were employed in 55 patients (98.2%) at baseline, with a mean daily dose of 43.04 ± 18.19 (range: 0 to 60) mg, decreasing to 8.95 ± 9.78 mg/day (range: 0 to 50) by week 48 (95% CI, 6.3 to 11.6). Sequential immunosuppressive therapies included mycophenolate mofetil (MMF) (7 cases, 12.5%), methotrexate (MTX) (2 cases, 3.6%), belimumab (2 cases, 3.6%), rituximab (1 case, 1.8%) and tocilizumab (1 case,1.8%). Most patients receiving MMF had relapsing or refractory disease. Of these, five were already on MMF at baseline, while two initiated MMF treatment later at week 4 and 12. Other medications, including MTX, belimumab, tocilizumab, and rituximab, were introduced at variable time points, mostly after week 12. Details on these therapies and corresponding outcomes are provided in **Table 2**. Besides, a total of 13 patients (23.2%) received a second dose of obinutuzumab at week 48, among whom 2 (15.4%) were re-treated due to disease flare, while the remaining 11 (84.6%) received the second dose as consolidation therapy.

**Table 2:** Sequential medications and treatment outcomes in SLE patients.

Sequential medications	Number of patients, n (%)	Disease stage, n (%)	Initiation week of medication, n (%)	Response at week 48, n (%)
Mycophenolate mofetil	7 (12.5)	New onset: 2 (28.6) Relapsing/Refractory:5 (71.4)	Week 0: 1 (14.3) Week 4: 1 (14.3) Week 12: 4 (57.1) Week 36: 1 (14.3)	Clinical remission: 2 (28.6) LLDAS^*^: 2 (28.6) Doris: 1 (14.3) Persistent: 2 (28.6)
Methotrexate	2 (3.6)	New onset: 1 (50.0) Relapsing/Refractory: 1 (50.0)	Week 0: 1 (50.0) Week 36: 1 (50.0)	Doris: 1 (50.0) LLDAS: 1 (50.0)
Belimumab	2 (3.6)	New onset: 2 (100.0)	Week 4: 1 (50.0) Week 12: 1 (50.0)	Clinical remission: 1 (50.0) LLDAS: 1 (50.0)
Tocilizumab	1 (1.8)	Relapsing/Refractory: 1 (100.0)	Week 12: 1 (100.0)	Clinical remission: 1(100.0)
Rituximab	1 (1.8)	Maintenance: 1 (100.0)	Week 36: 1 (100.0)	Doris: 1(100.0)

^*^To better illustrate the proportions, the number of patients achieving LLDAS in this table excludes those who met the DORIS remission criteria.

Obinutuzumab demonstrated a generally favorable safety profile in our study. Infections were the most common adverse events, with COVID-19 being the most frequent (9 cases, 16.1%), followed by pneumonia (6 cases, 10.7%), cytomegalovirus (3 cases, 5.4%), and influenza A/B virus (2 cases, 3.6%). Single cases of fungal infection and herpes zoster were also reported (each 1.8%). All infectious events resolved promptly with appropriate antibiotic or antiviral therapy. Infusion-related reactions were observed in 4 patients (7.1%), including vomiting and cutaneous pruritus, both occurred in 2 patients. No treatment-related deaths was observed (**Supplementary Table S2**).

## Discussion

While obinutuzumab has demonstrated promise in clinical trials such as NOBILITY and REGENCY (7, 8), its real-world application in SLE remains incompletely understood. Our study provides exploratory, real-world evidence describing treatment responses and safety outcomes in a heterogeneous SLE population. These data contribute to a more comprehensive understanding of obinutuzumab’s clinical utility beyond controlled experimental settings.

A key strength of our study lies in the inclusion of high-risk patients often excluded from randomized controlled trials (RCTs). Our cohort included 39.3% of the patients with high disease activity (SLEDAI-2K>12), encompassing severe manifestations including neuropsychiatric lupus, advanced renal impairment (eGFR <30 mL/min/1.73 m² or thrombotic microangiopathy requiring renal replacement therapy) and life-threatening thrombocytopenia (platelet count <30 × 10^9^/L). Notably, we also included patients with SLEDAI-2K ≤6 who presented with severe organ-specific pathology (e.g., autoimmune hemolytic anemia, gastrointestinal vasculitis) inadequately captured by standard activity indices. This inclusive approach enhances the real-world relevance of our findings and complements evidence from more selective RCT populations.

Despite the disease severity, 69.6% of patients achieved DORIS remission or LLDAS by week 48, with 19.6% remained clinical stable in our study. These outcomes suggest that obinutuzumab can be associated with meaningful disease improvement in difficult-to-treat populations.

In addition to the overall therapeutic benefit, organ-specific responses were particularly encouraging. Among patients with lupus nephritis, 86.7% achieved complete renal response by week 48—a rate exceeding those reported in the NOBILITY and REGENCY trials (7, 8). This superior outcome in our study is considered multifactorial, attributable to the smaller LN cohort size, shorter disease duration (median 2 months), fewer refractory cases, and differences in study design. Similarly, significant hematologic improvements were observed. Patients with AIHA and ITP experienced marked increases in hemoglobin and platelet counts. Although data from large trials remain limited, previous case reports have also suggested the utility of obinutuzumab in refractory autoimmune cytopenias (5, 19, 20). These findings support a potential role for obinutuzumab in controlling systemic disease while providing organ-specific benefits, particularly in renal and hematologic manifestations associated with poor prognosis. The impact of concomitant immunosuppressants on obinutuzumab’s treatment results is likely minimal, given that their use was limited to a small patient subset and initiation occurred long after obinutuzumab therapy in nearly half of the cases. Therefore, although baseline heterogeneity and adjunct therapies may have contributed to the improvements, our results remain clinically meaningful as they reflect real-world treatment outcomes in a challenging SLE population.

Obinutuzumab demonstrated a generally acceptable safety profile and was well-tolerated The most common adverse events (AEs) were infections, notably COVID-19 and pneumonia. While the pandemic context was a potential factor, profound B-cell depletion was also the likely contributor, and the risk may have been further influenced by prior and concomitant therapies, as most infections occurred in patients receiving multiple immunosuppressants or corticosteroids ≥10 mg/day. However, the limited sample size and relatively short follow-up period precluded establishing statistical significance for these observations and may have led to an underestimation of long-term risks. All infusion-related reactions, such as vomiting and pruritus, were predominantly mild. No treatment-related deaths occurred.

This study has several limitations. Its retrospective, single-center, non-randomized design, along with the use of concomitant treatments, may have introduced selection bias and residual confounding. The relatively short follow-up period may also have led to an underestimation of late relapses or delayed adverse events. Therefore, direct comparison of our outcomes with those reported in RCTs such as NOBILITY and REGENCY should be interpreted with caution, given differences in study design, patient selection, and baseline disease characteristics. In addition, the role of obinutuzumab in sequential therapy was only evaluated descriptively by assessing disease response after its introduction, as comparative analyses across different sequences were not feasible due to the small sample size and clinical heterogeneity. In light of these limitations, the findings should be considered as exploratory and hypothesis-generating rather than confirmatory. Future multicenter, prospective, randomized studies with longer follow-up are warranted to better evaluate the long-term efficacy and adverse events of obinutuzumab in SLE. Despite these constraints, our outcomes compare favorably with those from other real-world SLE cohorts (21–23), supporting the therapeutic potential of obinutuzumab, albeit with due consideration of differences in study design, patient selection, and concomitant therapies.

In conclusion, this real-world analysis suggests that a single obinutuzumab course may offer clinically meaningful disease control with acceptable safety in complex SLE cases, including severe/refractory manifestations. These findings expand current knowledge on the therapeutic potential of obinutuzumab and support its further systematic investigation.

## Data Availability

The original contributions presented in the study are included in the article/**Supplementary Material**. Further inquiries can be directed to the corresponding author.
