# Correction: Obinutuzumab in systemic lupus erythematosus: a real-world experience

**DOI:** 10.3389/fimmu.2025.1764419

**Published:** 2025-12-16

**Authors:** Chunmei Wu, Yinglu Wang, Shenshen Chen, Yanwei Lin, Fang Du, Xiaodong Wang, Sheng Chen, Liangjing Lu, Shuang Ye, Huihua Ding, Qiong Fu

**Affiliations:** 1 Department of Rheumatology, Renji Hospital, Shanghai Jiao Tong University School of Medicine, Shanghai, China; 2 Shanghai Immune Therapy Institute, Shanghai, China

**Keywords:** systemic lupus - erythematosus, obinutuzumab, biologics, real world study, B cell depletion

There was a mistake in **Table 1** as published. The percentage given for the ‘Relapsing’ column was erroneously written as “27 (55.4)”. This has been corrected to read “27 (48.2)”. The percentage given for the “musculoskeletal involvement” column was erroneously written as “9 (16.1)”. This has been corrected to read “10 (17.9)”. The corrected **Table 1** appears below.

**Table 1:** Baseline characteristics of SLE patients treated with obinutuzumab.

SLE Patients (n=56)	
Epidemiologic features	
Female, n (%)	45 (80.3)
Disease duration at first treatment, mean ± SD, (months)	48.52 ± 49.32 (0.3-168)
Disease state, n (%)	
New-onset	20 (35.7)
Relapsing	27 (48.2)
Refractory	4 (7.1)
Maintenance	5 (8.9)
Age at obinutuzumab treatment, mean ± SD, (years)	30.98 ± 12.45 (13-65)
Clinical features, n (%)	
Fever	15 (26.8)
Mucocutaneous involvement	20 (35.7)
Musculoskeletal involvement	10 (17.9)
Serositis	19 (33.9)
Hematologic abnormalities	30 (53.6)
Autoimmune hemolytic anemia	12 (21.4)
Hemoglobin level≦60g/L	3 (25.0)
Immune thrombocytopenia	15 (26.8)
Plate level≦30×10^9^/L	5 (33.3)
Leukocytopenia	5 (8.9)
Lupus nephritis	15 (26.8)
24-hour urine protein, mean ± SD, (g/24h)	2.28 ± 0.49 (0.67-6.09)
Serum creatinine, mean ± SD, (µmol/L)	83.83 ± 41.29 (32-185)
Neuropsychiatric lupus	18 (32.1)
Gastrointestinal vasculitis	5 (8.9)
Pulmonary hypertension	4 (7.1)
Thrombotic microangiopathy	4 (7.1)
Myocardial involvement	2 (3.6)
Antiphospholipid syndrome		
Deep venous thrombosis	7 (12.5)
Cerebral infraction	3 (5.4)
Pulmonary embolism	1 (1.8)
Serologic characterization	
WBC, mean ± SD, (×10^9^/L)	8.10 ± 4.29 (1.02-21.72)
Hb, mean ± SD, (g/L)	104.89 ± 26.06 (51.00-154.00)
PLT, mean ± SD, (×10^9^/L)	167.71 ± 120.57 (2.00-494.00)
C3, mean ± SD, (g/L)	0.65 ± 0.28 (0.19-1.34)
C4, mean ± SD, (g/L)	0.12 ± 0.10 (0.02-0.66)
IgA, mean ± SD, (g/L)	2.57 ± 1.26 (0.27-6.55)
IgM, mean ± SD, (g/L)	1.03 ± 0.70 (0.21-3.00)
IgG, mean ± SD, (g/L)	16.7 ± 8.9 (6.15-53.90)
Anti-dsDNA (ELASA), mean ± SD, (IU/mL)	167.90 ± 131.26 (22.46-505.06)
Anti-dsDNA (RIA), mean ± SD, (IU/mL)	51.66 ± 35.32 (4.96-100)
Antinuclear antibody positivity, n (%)	56 (100.0)
Anti-SSA positivity, n (%)	28 (50.0)
Anti-Ro52 positivity, n (%)	23 (41.1)
Anti-SSB positivity, n (%)	7 (12.5)
Anti-U1RNP positivity, n (%)	20 (35.7)
Anti-Sm positivity, n (%)	7 (12.5)
Anti-Rib-P positivity, n (%)	15 (26.8)
Anti-Histone positivity, n (%)	12 (21.4)
Antiphospholipid antibody positivity, n (%)	37 (66.1)
B lymphocyte levels	
B lymphocyte percentage, mean ± SD, (%)	17.76 ± 12.07 (1.62-44.98)
B lymphocyte count, mean ± SD, (cells/µL)	247.65 ± 293.74 (13.10-1268.40)
Disease activity	
SLEDAI-2K, mean ± SD	11.75 ± 9.31(0-46)
PGA, mean ± SD	1.66 ± 0.87 (0-3)
Previous treatments, n (%)	
Glucocorticoid	48 (85.7)
Hydroxychloroquine	41 (73.2)
Immunosuppressants	33 (58.9)
MMF	20 (35.7)
TAC	14 (25.0)
CTX	9 (16.1)
CsA	8 (14.3)
LEF	6 (10.7)
MTX	5 (8.9)
Tripterygium glycosides	1 (1.8)
AZA	1 (1.8)
Biologics	15 (26.8)
RTX	11 (19.6)
Belimumab	5 (8.9)
Telitacicept	2 (3.6)

SD, standard deviation; SEM, standard error of the mean; MMF, Mycophenolate mofetil; TAC, Tacrolimus; CTX, Cyclophosphamide; CsA, Cyclosporine A; LEF, Leflunomide; MTX, Methotrexate; AZA, Azathioprine; RTX, Rituximab; ELASA, Enzyme-Linked Immunosorbent Assay; RIA, Radioimmunoassay.

In the **Abstract**, two percentages were reported incorrectly in the first sentence of the **Results** section. This has been corrected to read:

“At baseline, 35.7% of patients were newly diagnosed, 55.4% had relapsing/refractory disease, and 8.9% were on maintenance therapy.”

A correction has been made to section **Results**, paragraph 2, to change “Besdieds” to “Besides”:

“Besides, antidsDNA antibody levels decreased, while complement levels exhibited a notable upward trend throughout the follow-up period.”

A correction has been made to section **Results**, paragraph 2, to change maitained” to “maintained”:

“Subgroups analysis revealed that all three subgroups, especially the new-onset and relapsing/refractory groups, showed a rapid decrease in SLEDAI-2K scores, which were maintained at low levels through week 48.”

The original version of this article has been updated.

