# Supplementary material for: Obinutuzumab in systemic lupus erythematosus: a real-world experience
Source: Front Immunol. 2025 Nov 19;16:1702550. doi: 10.3389/fimmu.2025.1702550 (PMC12672876; doi:10.3389/fimmu.2025.1702550)
Supplement: Supplementary file 1 [file Table1.docx]

**SUPPLEMENTARY MATERIAL FOR**

**Obinutuzumab in Systemic Lupus Erythematosus: a real-world experience**

Chunmei Wu,^1^† Yinglu Wang,^1^† Shenshen Chen, ^1^ Yanwei Lin, ^1^ Fang Du, ^1^ Xiaodong Wang, ^1^ Sheng Chen, ^1^ Liangjing Lu, ^1^ Shuang Ye, ^1^ Huihua Ding,^1*^ Qiong Fu^1,2*^

**The supplemental material includes:**

1. Supplementary Figure 1-3
2. Supplementary Table 1-2

**
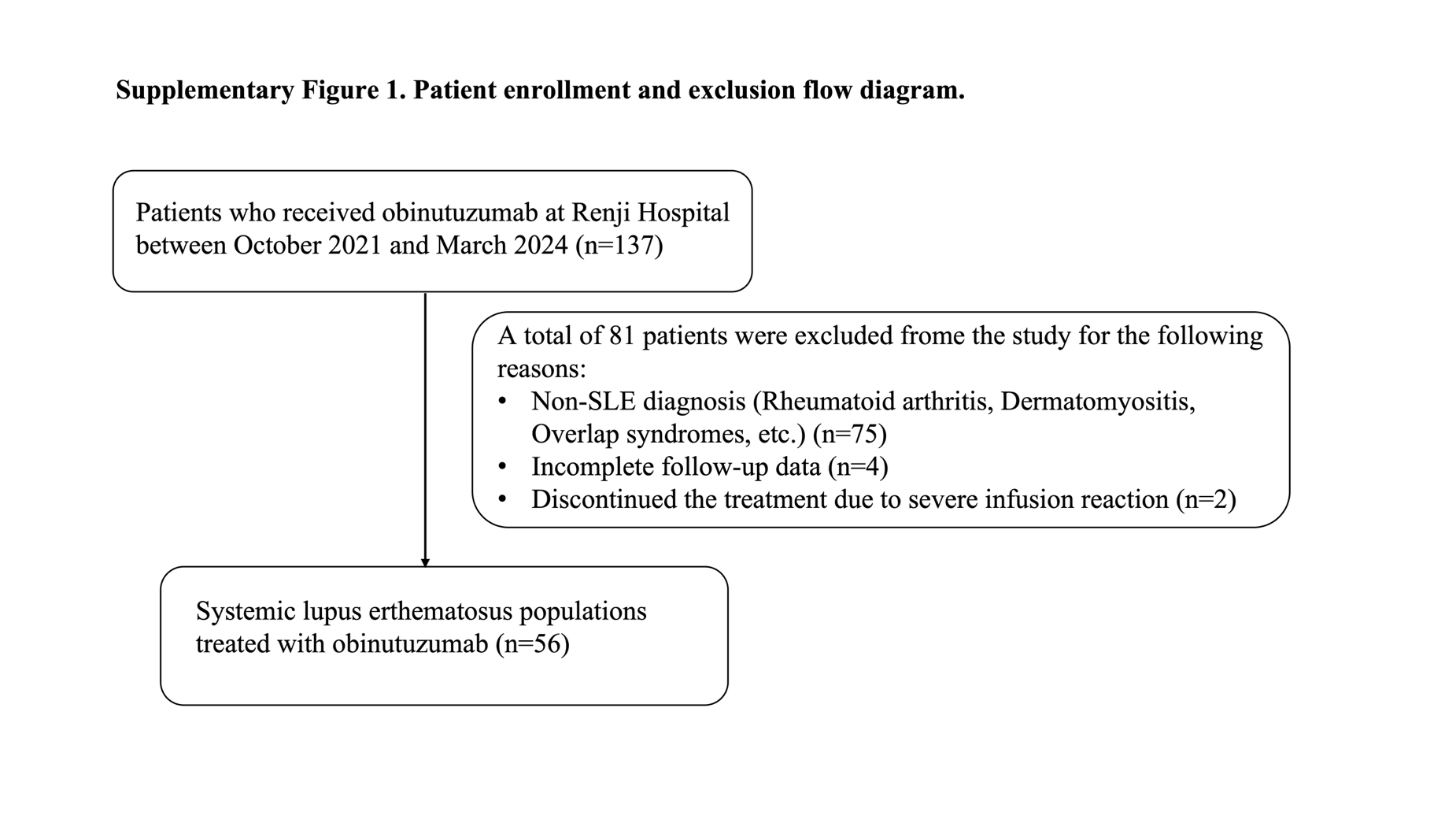
**

**Supplementary Figure 1.** Patient enrollment and exclusion flow diagram. A total of 137 patients who received obinutuzumab between October 2021 and March 2024 were screened. After excluding non-SLE cases (n=75), patients with incomplete follow-up data (n=4), and those who discontinued treatment due to severe infusion reactions (n=2), 56 patients were included in the final analysis.

**
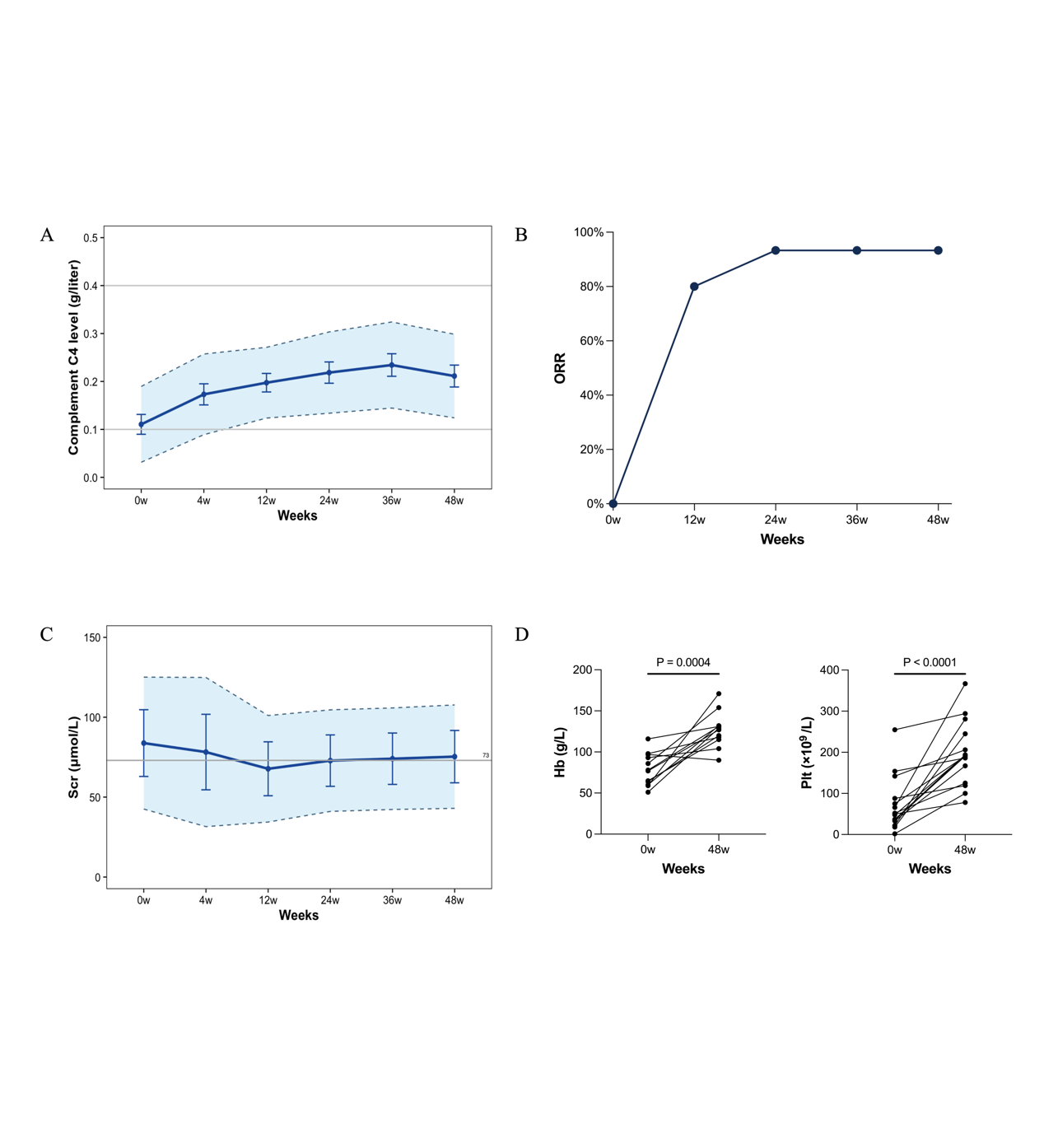
**

**Supplementary Figure 2.** Efficacy of obinutuzumab in improving renal and hematologic outcomes in SLE patients with organ-specific involvement. (A) Complement C4 changes during the 48-week follow-up. (B) The overall renal response (ORR) rate changes in patients with LN during follow-up. ORR was achieved in 80% of patients at week 12, 93.3% at week 24 and sustained this response through week 48. (C) Changes in serum creatinine before and after obinutuzumab treatment in LN patients. The solid lines represent the mean values, the error bars illustrate 95% confidence intervals (CIs), and the shaded areas indicate the standard deviation (SD). (D) Hemoglobin (Hb) levels (left) in 12 patients with autoimmune hemolytic anemia (AIHA) and Platelet (Plt) counts (right) in 15 patients with immune thrombocytopenia (ITP) significantly increased from baseline to week 48. *P* values are determined by paired *t* test in (D).

**
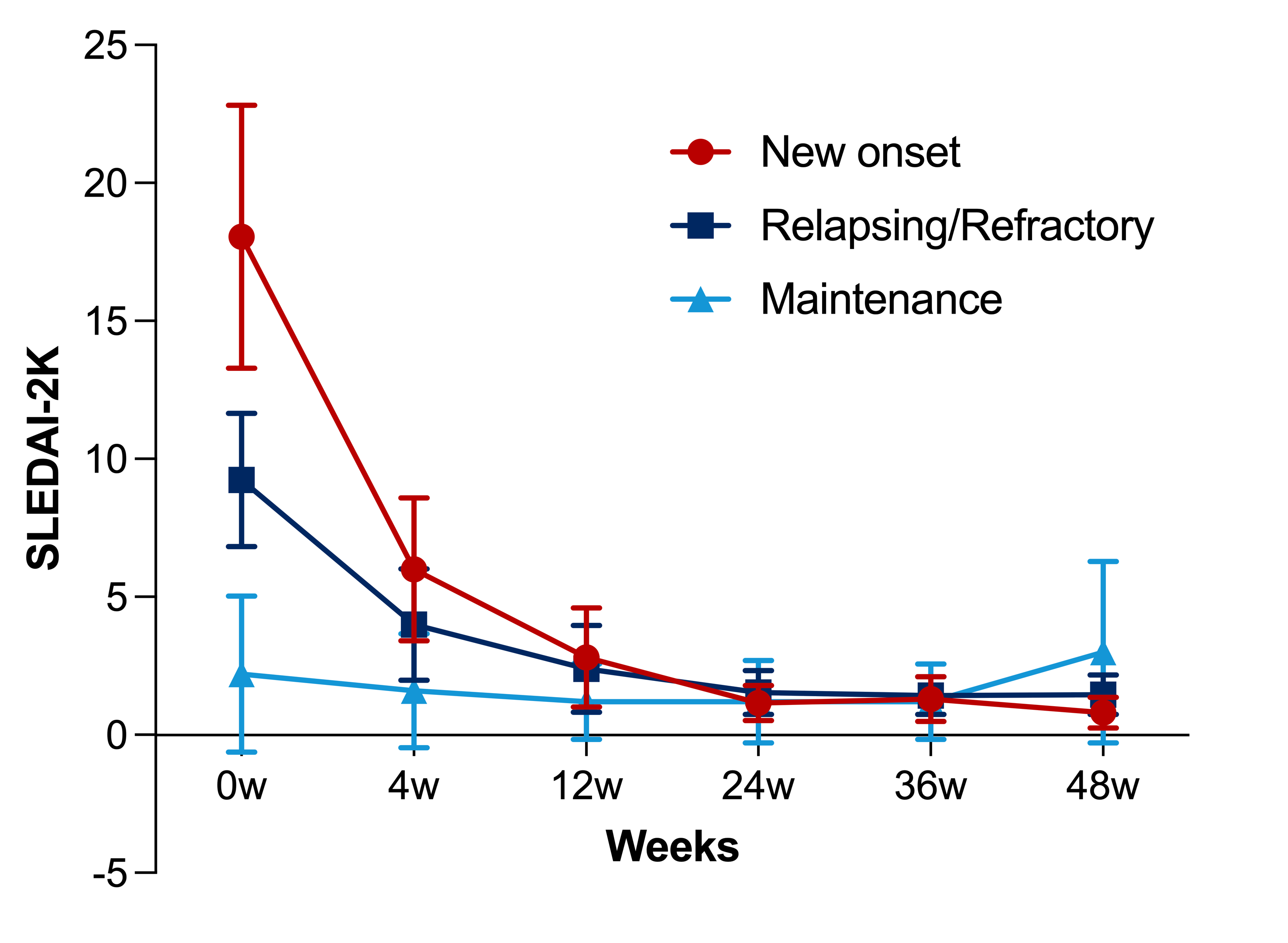
**

**Supplementary Figure 3.** Longitudinal changes in SLEDAI-2K scores among three different disease-stage subgroups during follow up**.** Mean SLEDAI-2K scores at baseline and follow-up visits (weeks 0, 4, 12, 24, 36, and 48) are shown for patients stratified by disease stage at treatment initiation (new-onset, relapsing/refractory, and maintenance).Solid lines represent mean values, and error bars indicate 95% confidence intervals (CIs). SLEDAI-2K scores declined rapidly across all subgroups, with the greatest reduction observed in the new-onset subgroup.

**Supplementary Table 1. Baseline characteristics across disease-stage subgroups.**

|  | Overall  (n=56) | New onset  (n=20) | Relapsing/Refractory  (n=31) | Maintenance  （n=5） |
| --- | --- | --- | --- | --- |
| Epidemiologic features |  |  |  |  |
| Female, n (%) | 45 (80.3) | 19 (95.0) | 21 (67.7) | 5 (100.0) |
| Disease duration at first treatment, mean ± SD, (months) | 48.52±49.32 (0.3-168) | 3.06±2.80 (0-12) | 73.73±47.80 (7-168) | 38.00±22.89 (12-72) |
| Age at obinutuzumab treatment, mean ± SD, (years) | 30.98±12.45 (13-65） | 30.45±15.43 (14-65) | 31.13±10.30 (16-52) | 32.20±14.18 (13-49) |
| Clinical features, n (%) |  |  |  |  |
| Fever | 15 (26.8) | 9 (45.0) | 6 (19.3) | 0 (0.0) |
| Mucocutaneous involvement | 20 (35.7) | 12 (60.0) | 8 (25.8) | 0 (0.0) |
| Musculoskeletal involvement | 9 (16.1) | 4 (20) | 5 (16.1) | 0 (0.0) |
| Serositis | 19 (33.9) | 12 (60.0) | 7 (22.6) | 0 (0.0) |
| Hematologic abnormalities | 30 (53.6) | 12 (60.0) | 18 (58.1) | 0 (0.0) |
| Autoimmune hemolytic anemia | 12 (21.4) | 5 (25.0) | 7 (22.6) | 0 (0.0) |
| Hemoglobin level≦60g/L | 3 (25.0) | 1 (20.0) | 2 (28.6) | NA |
| Immune thrombocytopenia | 15 (26.8) | 7 (35.0) | 8 (25.8) | 0 (0.0) |
| Plate level≦30×109/L | 5 (33.3) | 4 (57.1) | 1 (12.5) | NA |
| Leukocytopenia | 5 (8.9) | 3 (15.0) | 2 (6.4) | 0 (0.0) |
| Lupus nephritis | 15 (26.8) | 9 (45.0) | 6 (19.3) | 0 (0.0) |
| 24-hour urine protein, mean±SD, (g/24h) | 2.28±0.49 (0.67-6.09) | 2.18±2.06 (0.67-6.05) | 2.44±1.88 (0.85-6.09) | NA |
| Serum creatinine, mean±SD, (µmol/L) | 83.83±41.29 (32-185) | 76.36±23.73 (43-113) | 95.03± 60.17 (32.40-185.00) | NA |
| Neuropsychiatric lupus | 18 (32.1) | 9 (45.0) | 7 (22.6) | 2 (40.0) |
| Gastrointestinal vasculitis | 5 (8.9) | 1 (5.0) | 4 (12.9) | 0 (0.0) |
| Pulmonary hypertension | 4 (7.1) | 2 (10.0) | 2 (6.4) | 0 (0.0) |
| Thrombotic microangiopathy | 4 (7.1) | 1 (5.0) | 3 (9.6) | 0 (0.0) |
| Myocardial involvement | 2 (3.6) | 1 (5.0) | 0 (0.0) | 1 (20.0) |
| Antiphospholipid syndrome |  |  |  |  |
| Deep venous thrombosis | 7 (12.5) | 0 (0.0) | 6 (19.3) | 1 (20.0) |
| Cerebral infraction | 3 (5.4) | 1 (5.0) | 1 (3.23) | 1 (20.0) |
| Pulmonary embolism | 1 (1.8) | 0 (0.0) | 1 (3.23) | 0 (0.0) |
| Serologic characterization |  |  |  |  |
| WBC, mean±SD, (×109/L) | 8.10±4.29 (1.02-21.72) | 7.77±4.57 (1.02-15.92) | 8.81±4.28 (2.1-21.72) | 4.96±0.37 (4.49-5.46) |
| Hb, mean±SD, (g/L) | 104.89±26.06 (51.00-154.00) | 91.25±22.59 (51-138) | 111.19±26.35 (56-154) | 120.40±12.86 (108-141) |
| PLT, mean±SD, (×109/L) | 167.71±120.57 (2.00-494.00) | 156.6±109.25 (2-401) | 173.42±135.49 (2-494) | 176.80±67.36 (88-250) |
| C3, mean±SD, (g/L) | 0.65±0.28 (0.19-1.34) | 0.51±0.29 (0.19-1.34) | 0.71±0.25 (0.22-1.21) | 0.83±1.33 (0.64-0.96) |
| C4, mean±SD, (g/L) | 0.12±0.10 (0.02-0.66) | 0.11±0.15 (0.20-0.66) | 0.12±0.08 (0.03-0.35) | 0.12±0.05 (0.07-0.19) |
| IgA, mean±SD, (g/L) | 2.57±1.26 (0.27-6.55) | 2.75±1.11 (0.89-5.90) | 2.53±1.41 (0/27-6.55) | 2.16±0.83 (0.82-2.99) |
| IgM, mean±SD, (g/L) | 1.03±0.70 (0.21-3.00) | 1.42±0.74 (0.37-3.00) | 0.87±0.60 (0.21-2.56) | 0.47±0.11 (0.38-0.63) |
| IgG, mean±SD, (g/L) | 16.7±8.9 (6.15-53.90) | 22.06±8.41 (9.82-39.10) | 13.84±8.41 (6.15-53.90) | 13.14±2.28 (10-16) |
| Anti-dsDNA (ELASA), mean±SD, (IU/mL) | 167.90±131.26 (22.46-505.06) | 199.82±135.10 (30.89-505.06) | 154.88±127.26 (22.46-411.75) | 120.91±139.90 (25.39-364.26) |
| Anti-dsDNA (RIA), mean±SD, (IU/mL) | 51.66 ± 35.32 (4.96-100) | 52.46±32.21 (11-100) | 53.11±37.59 (6.99-100) | 41.03±37.24 (4.96-100) |
| Antinuclear antibody positivity, n (%) | 56 (100.0) | 20 (100.0) | 31 (100.0) | 5 (100.0) |
| Anti-SSA positivity, n (%) | 28 (50.0) | 13 (65.0) | 12 (38.7) | 3 (60.0) |
| Anti-Ro52 positivity, n (%) | 23 (41.1) | 10 (50.0) | 11 (35.4) | 2 (40.0) |
| Anti-SSB positivity, n (%) | 7 (12.5) | 2 (10.0) | 4 (12.9) | 1 (20.0) |
| Anti-U1RNP positivity, n (%) | 20 (35.7) | 5 (25.0) | 15 (48.3) | 0 (0.0) |
| Anti-Sm positivity, n (%) | 7 (12.5) | 5 (25.0) | 2 (6.4) | 0 (0.0) |
| Anti-Rib-P positivity, n (%) | 15 (26.8) | 9 (45) | 6 (19.3) | 0 (0.0) |
| Anti-Histone positivity, n (%) | 12 (21.4) | 3 (15.0) | 8 (25.8) | 1 (20.0) |
| Antiphospholipid antibody positivity, n (%) | 37 (66.1) | 15 (75.0) | 20 (64.5) | 2 (40.0) |
| B lymphocyte levels |  |  |  |  |
| B lymphocyte percentage, mean±SD, (%) | 17.76±12.07 (1.62-44.98) | 22.63±9.58 (11.50-44.98) | 15.70±13.12 (0.40-44.20) | 7.64±6.12 (1.62-17.62) |
| B lymphocyte count, mean±SD, (cells/µL) | 247.65±293.74(13.10-1268.40) | 313.95±365.55 (36.6-1268.40) | 229.48±254.71 (2.2-947.40) | 95.12±72.61 (20.9-174.6) |
| Disease activity |  |  |  |  |
| SLEDAI-2K, mean±SD | 11.75±9.31(0-46) | 18.05±10.20 (4-46) | 9.23±6.61 (1-26) | 2.20±2.28 (0-5) |
| PGA, mean±SD | 1.66±0.87 (0-3) | 2.21±0.74 (0.5-3) | 1.51±0.74 (0.5-2.8) | 0.38±0.22 (0-0.5) |
| Previous treatments, n (%) |  |  |  |  |
| Glucocorticoid | 48 (85.7) | 12 (60.0) | 31 (100) | 5 (100.0) |
| Hydroxychloroquine | 41 (73.2) | 10 (50.0) | 26 (83.9) | 5 (100.0) |
| Immunosuppressants | 33 (58.9) | 3 (15.0) | 27 (87.1) | 3 (60.0) |
| MMF | 20 (35.7) | 1 (3.2) | 16 (51.6) | 3 (60.0) |
| TAC | 14 (25.0) | 2 (6.5) | 10 (32.3) | 2 (40.0) |
| CTX | 9 (16.1) | 0 (0.0) | 9 (29.0) | 0 (0.0) |
| CsA | 8 (14.3) | 0 (0.0) | 7 (22.6) | 1 (20.0) |
| LEF | 6 (10.7) | 0 (0.0) | 5 (16.1) | 1 (20.0) |
| MTX | 5 (8.9) | 0 (0.0) | 5 (16.1) | 0 (0.0) |
| Tripterygium glycosides | 1 (1.8) | 0 (0.0) | 1 (3.2) | 0 (0.0) |
| AZA | 1 (1.8) | 0 (0.0) | 1 (3.2) | 0 (0.0) |
| Biologics | 15 (26.8) | 1 (3.2) | 9 (29.0) | 5 (100.0) |
| RTX | 11 (19.6) | 0 (0.0) | 6 (19.4) | 5 (100.0) |
| Belimumab | 5 (8.9) | 0 (0.0) | 4 (12.9) | 1 (20.0) |
| Telitacicept | 2 (3.6) | 1 (3.2) | 1 (3.2) | 0 (0.0) |

SD, standard deviation; SEM, standard error of the mean; MMF, Mycophenolate mofetil; TAC, Tacrolimus; CTX, Cyclophosphamide; CsA, Cyclosporine A; LEF, Leflunomide; MTX, Methotrexate; AZA, Azathioprine; RTX, Rituximab; ELASA, Enzyme-Linked Immunosorbent Assay; RIA, Radioimmunoassay.

**Supplementary Table 2.** Infusion-Related Adverse Events with Obinutuzumab.

| Adverse Events | Case | Occurrence Rate (%) |
| --- | --- | --- |
| Infections | 14 | 25.0 |
| Covid-19 | 9 | 16.1 |
| Pneumonia | 6 | 10.7 |
| Cytomegalovirus | 3 | 5.4 |
| Influenza A/B Virus | 2 | 3.6 |
| Fungus | 1 | 1.8 |
| Herpes Zoster | 1 | 1.8 |
| Infusion Reactions | 4 | 7.1 |
| Vomit | 2 | 3.6 |
| Cutaneous Pruritus | 2 | 3.6 |
